# Computational Multiscale Study of the Interaction Between the PDMS Polymer and Sunscreen-Related Pollutant Molecules

**DOI:** 10.3390/molecules29204908

**Published:** 2024-10-17

**Authors:** Stevan Armaković, Đorđe Vujić, Boris Brkić

**Affiliations:** 1 University of Novi Sad, Faculty of Sciences, Department of Physics, Trg D. Obradovića 4, 21000 Novi Sad, Serbia; 2 BioSense Institute, University of Novi Sad, Dr Zorana Djindjića 1, 21000 Novi Sad, Serbia; d.vujic@biosense.rs

**Keywords:** MIMS, membranes, PDMS, GFN2-xTB, MD, DFT

## Abstract

Sunscreen molecules play a critical role in protecting skin from ultraviolet radiation, yet their efficient detection and separation pose challenges in environmental and analytical contexts. In this work, we employ a multilevel modeling approach to investigate the molecular interactions between representative sunscreen molecules and the polydimethylsiloxane (PDMS) polymer, a material widely recognized for its sorbent properties. Our goal is to explore how these interactions can be fine-tuned to facilitate the effective separation of sunscreen molecules in portable membrane inlet mass spectrometry (MIMS) systems, potentially leading to the development of new membrane materials. Using a combination of advanced computational techniques—force field molecular dynamics simulations, semiempirical GFN2-xTB, and density functional theory calculations—we assess the interaction strength and noncovalent interactions of sunscreen molecules, namely oxybenzone, naphthalene, benzo[a]anthracene, avobenzone, and 1,3,5-trichlorobenzene, with PDMS. Additionally, the effect of temperature on the interaction dynamics is evaluated, with the aim of extending the sorbent capacities of PDMS beyond light polar molecules to larger, polar sunscreen compounds. This study provides critical insights into the molecular-level interactions that may guide the design of novel membrane materials for efficient molecular separation.

## 1. Introduction

**Figure 1:**
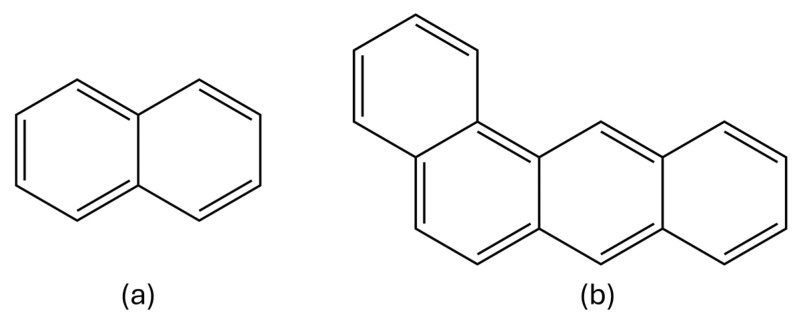
Structures of selected PAHs: (**a**) naphthalene, (**b**) benzo[a]anthracene.

Water pollution, caused by the discharge of harmful substances into rivers, lakes, and oceans, is one of the most urgent environmental challenges of our time. From industrial waste to agricultural runoff, the sources of pollutants are diverse and widespread. Among those pollutants, frequent compound classes are polyaromatic hydrocarbons (PAHs), while sunscreen-agent-based pollutants are prevalent in popular tourist resorts. Pollution monitoring is important from the perspective of public health and environmental protection. PAHs belong to the group of semi-volatile compounds that share one chemical feature among themselves: the presence of two or more aromatic rings in their structure [1]. Besides natural sources, which are almost negligible in terms of output, the majority of PAH sources are related to human activity, such as industry, agriculture, and motor vehicles [1]. A wide variety of health hazards emerge from exposure to PAHs, such as cancer, cardiovascular diseases, respiratory issues, and developmental effects [1,2,3]. This paper will focus on naphthalene and benzo[a]anthracene, shown in Figure 1.

**Figure 2:**
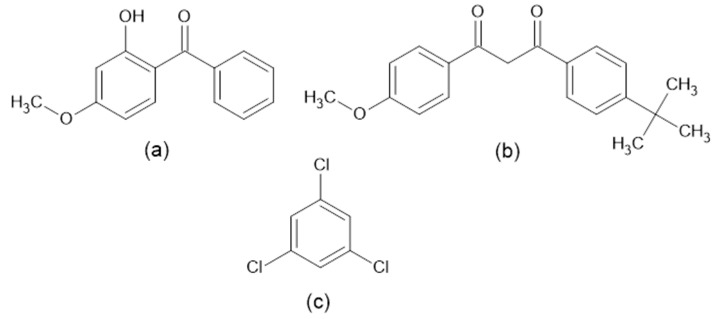
Structures of selected sunscreen agents: (**a**) oxybenzone, (**b**) avobenzone, and structure of 1,3,5-trichlorobenzene (**c**).

Regarding sunscreen agents, their toxicity and adverse health effects have only relatively recently come to attention, and they are being considered as emerging pollutants since their use has been constantly increasing over the last few decades [4]. Adverse effects are being studied extensively, with many authors reporting their possible relation to skin cancer [5,6,7]. It has been proven that some of them, such as oxybenzone, are found in human urine and breast milk [6], which is a clear indication of their ability to penetrate the skin and be absorbed into the body. Since there are more than a dozen chemicals used as blockers of UV light in sunscreen agents, our research will be narrowed down to two chemicals, which are avobenzone and oxybenzone. Their structures are represented in Figure 2a and Figure 2b, respectively.

In this work, we will examine the keto form and two possible enol forms of avobenzone. In these enol forms, a hydroxyl group (OH) replaces the oxygen atom (O) in the central part of the molecule, resulting in two distinct configurations [8].

The last compound that does not belong to any of those previously mentioned and that will be the subject of our research is 1,3,5-trichlorobenzene, whose structure is presented in Figure 2c. The Agency for Toxic Substances and Disease Registry reports that trichlorobenzenes are mostly used as solvents and intermediary compounds in chemical industries. Acute exposure can lead to respiratory irritation, eye and skin irritation, and neurological effects [9,10].

As all of the mentioned compounds are recognized as health hazards, they are subject to monitoring and regulation under various EU directives. Sunscreen agents are monitored in accordance with the EU Commission Implementing Decision (EU) 2022/1307 of 22 July 2022, which includes them on the surface water watch list [11]. Polyaromatic hydrocarbons (PAHs), on the other hand, are regulated under the EU Directive 2013/39/EU, which establishes environmental quality standards for priority substances and other pollutants in water [12].

**Figure 3:**
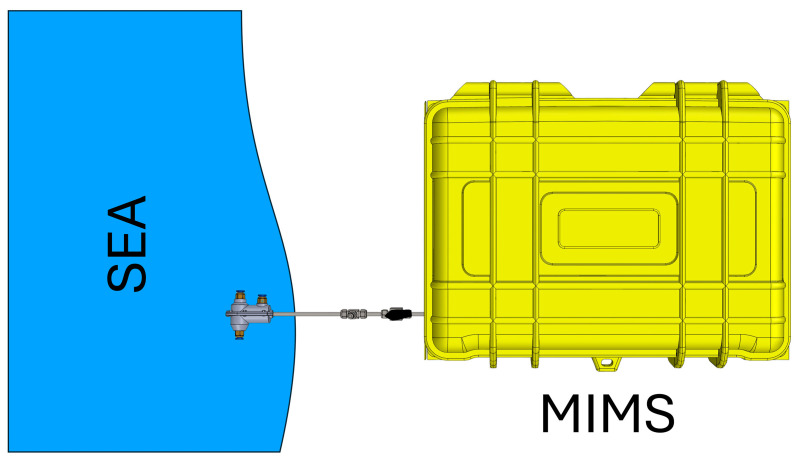
Schematic diagram for in situ sampling of seawater using MIMS.

The analysis of polycyclic aromatic hydrocarbons (PAHs) and chlorinated hydrocarbons, as well as many other volatile organic compounds (VOCs), and semi-VOCs is commonly performed using commercial techniques such as gas chromatography-mass spectrometry (GC-MS) or high-performance liquid chromatography (HPLC) [13,14]. These methods are favored for their high accuracy, repeatability, and low limits of detection (LOD). However, despite advantages, these techniques have certain limitations. They are time-consuming, laboratories are often at significant distances from the sampling site, and it is impossible to perform real-time analysis and extensive sample preparation [15,16]. The demand for fast, reliable, and real-time analysis, as well as the need to overcome the limitations of traditional analytical methods, has led to the development of membrane inlet mass spectrometry (MIMS) [17,18]. MIMS offers several significant advantages over conventional techniques such as GC-MS and HPLC. The most notable benefit of the MIMS system is its ability to perform analyses without the need for extensive sample preparation, which not only saves time but also reduces the potential for sample contamination and loss [19]. Furthermore, the MIMS system is particularly well-suited for in situ analysis, making it a versatile tool for field applications. Its portability and battery-powered operation allow for deployment in remote or challenging environments where traditional laboratory equipment would be impractical. This capability ensures that data can be collected and analyzed directly at the sampling site, providing real-time results that are critical for timely decision making in environmental monitoring, industrial process control, and other applications requiring immediate feedback [20,21]. Thus, MIMS represents a valuable advancement in analytical technology, addressing both the speed and logistical challenges associated with conventional methods. Figure 3 shows a schematic diagram for in situ sampling of seawater using MIMS.

Limitations of the MIMS system can be addressed by developing innovative membrane designs and modifying experimental parameters, such as temperature and water flow rates. MIMS has been demonstrated to effectively detect small polar molecules, including acetone and C1-C5 alcohols [22] and a variety of non-polar volatile and semi-volatile molecules. Since PAHs and sunscreen agents are currently undetectable by MIMS, it is assumed that their bonding energies with PDMS (polydimethylsiloxane) are too high, preventing their passage through the membrane. The hypothesis of this study is that one of the key factors influencing the interaction between a membrane and a molecule is the bonding energy between them since the molecules that can pass through the PDMS membranes could be detected by the mass spectrometer. The objective of this research is to evaluate the bonding energies of interactions between PDMS membranes and the specified molecules, and then compare these values with molecules that can be detected, such as benzene, toluene, xylene, 1,2-dichloroethane, and tetrachloroethylene.

Atomistic calculations are an invaluable tool in computational science, offering deep insights into the atomic-scale behavior of materials and molecules. The possibilities of atomistic calculations are critical for predicting the properties and interactions of systems before experimental synthesis, saving both time and resources in the research and development process [23,24]. Atomistic simulations combined with experimental results provide a comprehensive understanding of complex phenomena, making them fundamental for exploring new materials and molecules [25,26,27,28]. The flexibility of available theoretical approaches allows researchers to investigate a wide array of atomic mechanisms with exceptional precision [29,30,31].

Herein, we utilized a suite of computational techniques to explore the molecular interactions between sunscreen molecules and the PDMS polymer, focusing on how these interactions can be fine-tuned for efficient separation. PDMS is well-regarded for its suitability in sorbing light polar molecules, but its ability to separate larger, polar sunscreen compounds is limited due to the strong interactions that trap these molecules. This presents a significant obstacle in mass spectrometric applications, where efficient separation is key for accurate analysis.

The primary goal of this study was to first identify the critical value of interaction energy suitable for filtration of selected sunscreen molecules, and then to investigate how the strong interaction between larger polar sunscreen molecules and PDMS could be reduced, aiming to improve the polymer’s suitability as an inlet material in separation systems. Our main strategy to achieve this was via the modulation of the system temperature.

We investigated a range of PAHs and sunscreen molecules to examine how molecular size and mass influence their interactions with PDMS. By studying the interactions between PDMS and smaller, light molecules—those that PDMS can easily filter—we were able to establish baseline interaction energies. These reference values serve as targets for achieving efficient separation through modifications such as temperature adjustment or alternative strategies.

Ultimately, a detailed analysis of the ground-state geometries of the intermolecular systems, combined with binding energies calculated using the GFN2-xTB method and the identification of noncovalent interactions via DFT, provided instructions as to how PDMS could be optimized for separating larger sunscreen molecules. This work highlights the potential of PDMS as a sorbent material, particularly when considering its modification to enhance separation efficiency for more complex, larger molecules.

## 2. Results and Discussion

### 2.1. Molecular Structures and Computational Workflow

In this study, we investigated three distinct types of molecular systems: (a) individual PAHs, with their structures depicted in Figure 1a, (b) intermolecular systems consisting of a single PDMS polymer chain and one PAH molecule, and (c) molecular dynamics systems containing 19 PDMS polymer chains interacting with a single PAH molecule. The geometrically optimized structures of the PAHs are shown in Figure S1 of the Supplementary Materials, while Table 1 provides an overview of all the systems studied in this work.

**Figure 4:**
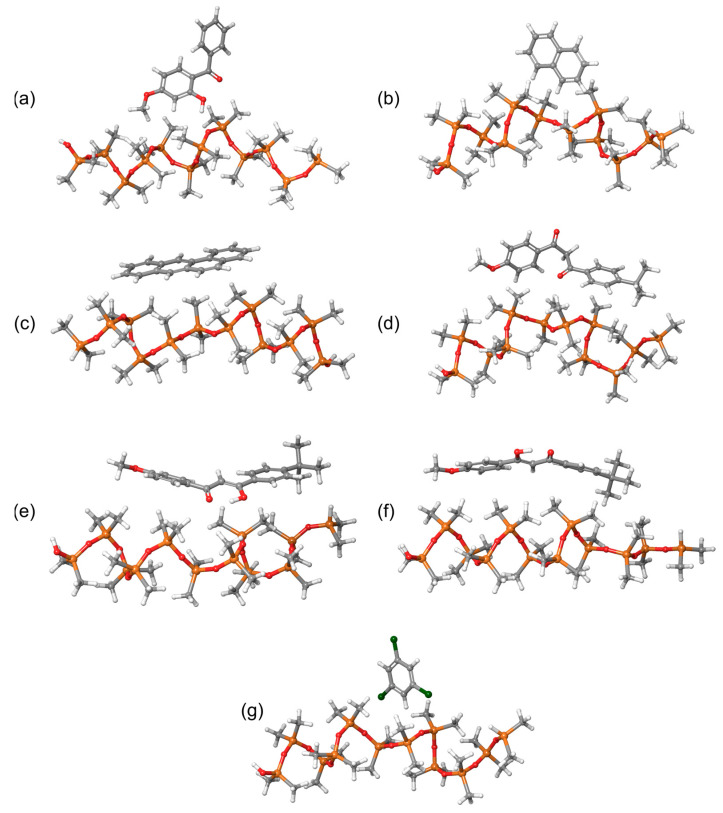
Intermolecular systems consisting of a PDMS chain and (**a**) oxybenzone, (**b**) naphthalene, (**c**) benzo[a]anthracene, (**d**) avobenzone (keto form), (**e**) avobenzone (enol1 form), (**f**) avobenzone (enol2 form) and (**g**) 1,3,5-trichlorobenzene sunscreen molecules.

Individual molecules were initially pre-optimized using the GFN2-xTB method with the xtb program, available through the atomistica.online molecular modeling platform. These pre-optimized structures were then re-optimized at the DFT level, employing the B3LYP/6-31G(d,p) level of theory. Following this, we generated intermolecular systems to serve as the basis for calculating binding energies using the GFN2-xTB method. In total, 11 intermolecular systems were examined in this study. For clarity, Figure 4 presents only the intermolecular systems involving PDMS polymer chains and sunscreen molecules, as these are the most relevant for designing sorbent materials suitable for detecting sunscreen compounds.

**Figure 5:**
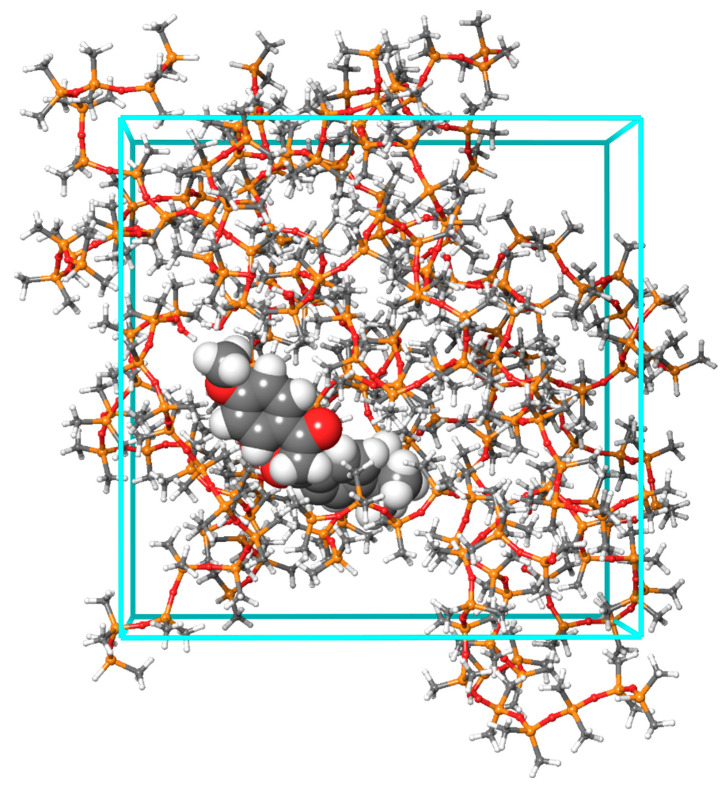
Representative MD system (19 PDMS chains and avobenzone)—PDMS chains are provided in ball-and-stick representation, while avobenzone is given in CPK representation.

The final step in structure generation was to create systems for molecular dynamics (MD) simulations, which closely mimic real-world conditions, where a single sunscreen molecule is embedded within a PDMS polymer medium. These MD systems, consisting of 19 PDMS chains and one PAH or sunscreen molecule, were constructed using the Packmol program [32,33], as implemented in the online version of the atomistica.online project [34,35]. The systems were then subjected to MD simulations using the OPLS3e force field within the Desmond program. A representative system, featuring 19 PDMS chains and an avobenzone (keto form) sunscreen molecule, is illustrated in Figure 5.

The primary goal was to evaluate the strength of interaction between PDMS and molecules detectable via the MIMS system, and to compare these interactions with those involving PDMS and sunscreen pollutant molecules. This comparison aimed to determine the threshold binding values at which PDMS can effectively function as a sorbent. By analyzing these values, we can identify the target interaction energies required when employing different or modified materials for MIMS sample probes.

The interaction strength was evaluated using two computational approaches: GFN2-xTB and force field-based MD simulations. The GFN2-xTB method was applied to systems containing a single PDMS chain and one molecule. While this approach may initially seem an approximation—considering that a polymer medium surrounds molecules in reality—it produced results consistent with experimental observations.

In this study, we assert that the strength of interaction fundamentally dictates the ability of the PDMS membrane or sorbent to effectively filter the desired compound and guide it toward the sensing system. Additionally, the size of the molecules plays a significant role, which is further explored in the subsequent section in relation to our findings.

### 2.2. MD Simulations—Interaction Energies

**Figure 6:**
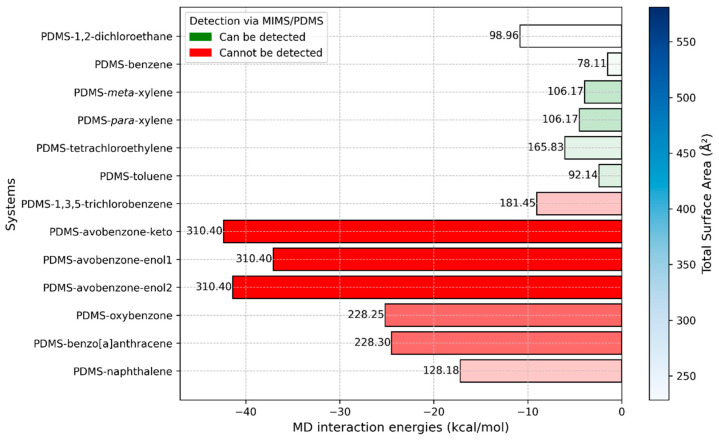
Multivariable plot of interaction energies against molecular weight and total surface area, obtained via MD simulations. The numbers on top of the bins represent molecular weight. The intensity of the blue color in the legend indicates the value of the total surface area and is directly correlated to the color intensities of bins. Red bins are related to compounds that current MIMS devices cannot detect, while green bins are related to compounds that can be detected.

We begin by discussing the results of the MD simulations, as these systems most closely resemble the real-world conditions encountered during measurements with the MIMS system, where the PDMS polymer medium almost entirely surrounds the sunscreen molecule. The interaction energies between the sunscreen molecules and the PDMS medium are presented in Figure 6, along with additional information about the molecules. This additional data is essential for providing a comprehensive explanation of the observed and calculated results.

In Figure 6, we present a multivariable plot of the interaction energies obtained from MD simulations. While the interaction strength between PDMS and the molecules primarily dictates whether the molecules can pass through the membrane and be filtered, the molecular size also plays a crucial role in determining the ease with which they can move through the polymer medium. To capture this, we included molecular weight and total surface area (TSA) for each molecule interacting with the PDMS polymer. Molecular weight is displayed at the top of the bins, while TSA values are indicated by a color gradient, with darker shades representing higher TSA values.

As previously mentioned, experimental results have shown that smaller molecules such as 1,2-dichloroethane, benzene, xylene, toluene, and tetrachloroethylene can be effectively filtered using PDMS membranes. This finding aligns well with the interaction energy values obtained from our MD simulations. As shown in Figure 6, these molecules exhibit significantly weaker interaction energies with the PDMS polymer medium compared to larger sunscreen pollutant molecules. However, there is one notable exception that can be explained by the size of the molecule. The interaction energy between PDMS and 1,2-dichloroethane is −10.82 kcal/mol, which is higher than the interaction energy between PDMS and 1,3,5-trichlorobenzene (−9.03 kcal/mol), a molecule that is more challenging to detect. Despite its stronger interaction energy, 1,2-dichloroethane is a tiny molecule as indicated by its molecular weight and TSA values, allowing it to penetrate and move through the PDMS polymer easily. This explains why it can still be readily filtered, despite its slightly stronger interaction with PDMS.

The cases of 1,2-dichloroethane and 1,3,5-trichlorobenzene interactions with PDMS should be particularly emphasized. Despite having very similar interaction energies with PDMS (−10.82 kcal/mol and −9.02 kcal/mol, respectively), 1,2-dichloroethane can be filtered, while 1,3,5-trichlorobenzene cannot. This suggests that interaction energy around −10 kcal/mol, as observed in our MD simulations, could represent a critical threshold for determining whether the PDMS polymer medium can filter a molecule. In other words, for effective filtration, the interaction energy between the compound and PDMS should be reduced to approximately −10 kcal/mol. Potential strategies to achieve this include manipulating the temperature, adding specific components to the system, or modifying the polymer with functional groups.

### 2.3. MD Simulations—Temperature Effects on the Interaction Strength

**Figure 7:**
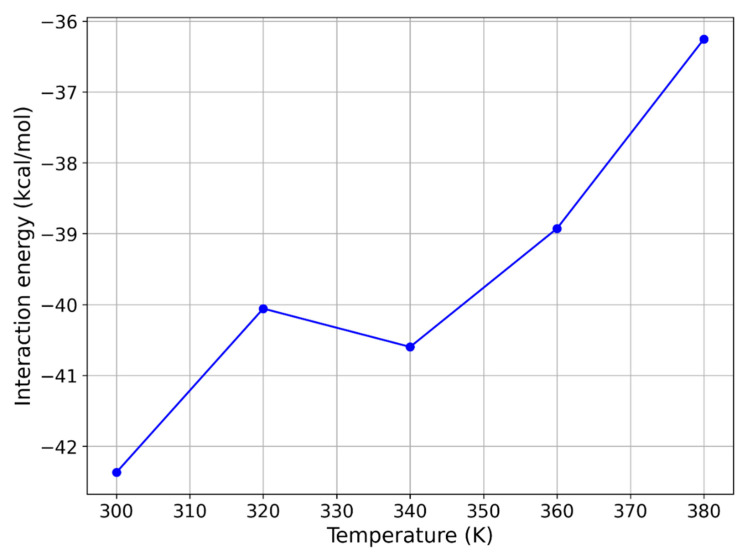
Temperature dependence of MD interaction energy of avobenzone keto form with PDMS.

As previously discussed, one of the approaches to manipulating the interaction energy between PDMS and a given molecule is to adjust the temperature. In one of our recent studies [36], it was shown that increasing the temperature can reduce the interaction energy between PDMS and volatile organic compounds (VOCs) such as bee alarm pheromones. This same strategy has been applied in the current study, beginning with an exploration of the effect of temperature on the interaction energy between PDMS and avobenzone, in particular its keto form. Avobenzone was chosen for two key reasons: first, it is one of the most commonly used UV-blocking agents in sunscreen products, and second, our calculations, as shown in Figure 7, indicate that the keto form of avobenzone exhibits powerful interactions with the PDMS polymer chain.

Given that temperature can be effectively controlled within MIMS systems, we first examined how increasing the temperature from 300 K to 380 K, in increments of 20 K, impacted the interaction energy between PDMS and avobenzone. For this detailed exploration, we focused on the keto form of avobenzone, as it exhibited the strongest interaction with PDMS. At 300 K, the interaction energy was highest for the keto form, followed by the enol1 and enol2 forms. The temperature dependence of the interaction energy for the keto form of avobenzone is illustrated in Figure 7.

The results shown in Figure 7 indicate that increasing the temperature significantly reduces the interaction energy between the keto form of avobenzone and the PDMS polymer medium. Specifically, the magnitude of the interaction energy steadily decreases as the temperature rises, reaching −36.254 kcal/mol at 380 K. Although this reduction of approximately 15% is not sufficient to bring the interaction energy into the desired range of 10 to 20 kcal/mol, it represents a meaningful decrease and a clear justification to test the same approach with other molecules.

**Figure 8:**
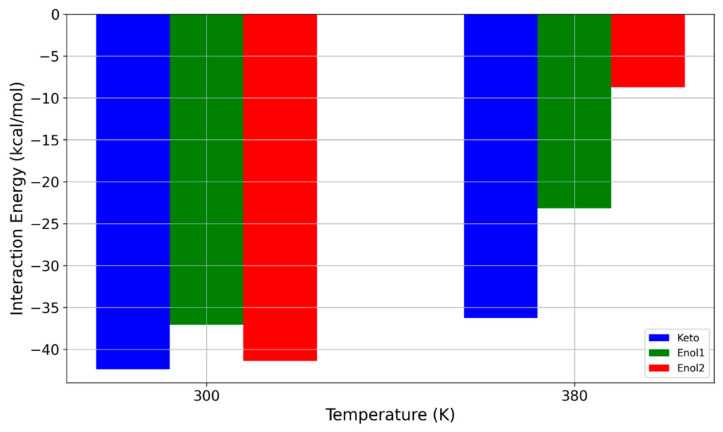
Influence of temperature on interaction energy between PDMS polymer and different forms of avobenzone.

In the next step, we examined the differences in interaction energies between the keto and enol forms of avobenzone when interacting with the PDMS polymer medium. Both forms are important for this study: the keto form plays a critical role in sunscreen formulations, while the enol forms are more prevalent in water and generally in nonpolar environments. The interaction energy results for both forms at two temperatures, 300 K and 380 K, are presented in Figure 8.

The results shown in Figure 8 suggest that the interaction between the PDMS polymer and the enol forms of avobenzone is even more sensitive to temperature changes compared to the keto form. While the differences in interaction energy at 300 K are not substantial, at 380 K, the interaction energies of the enol forms with PDMS are significantly lower than those of the keto form. Specifically, for the enol1 form, the interaction energy decreases by nearly 40%, from −37.075 kcal/mol at 300 K to −23.177 kcal/mol at 380 K. A more pronounced reduction occurs for the enol2 form, where increasing the temperature by 80 K results in an almost 80% decrease in interaction energy, dropping from −41.377 kcal/mol to −8.741 kcal/mol.

The remarkable drop in interaction energy strength to below −10 kcal/mol at higher temperatures suggests that, under these conditions, even avobenzone (enol form which is expected in water) could be filtered through a PDMS-based membrane due to the temperature increase alone. The dynamics of the hydroxyl group in the central part of the avobenzone molecule likely play a critical role in this significant decrease in interaction energy. This phenomenon will be explored in greater detail in the following section.

**Figure 9:**
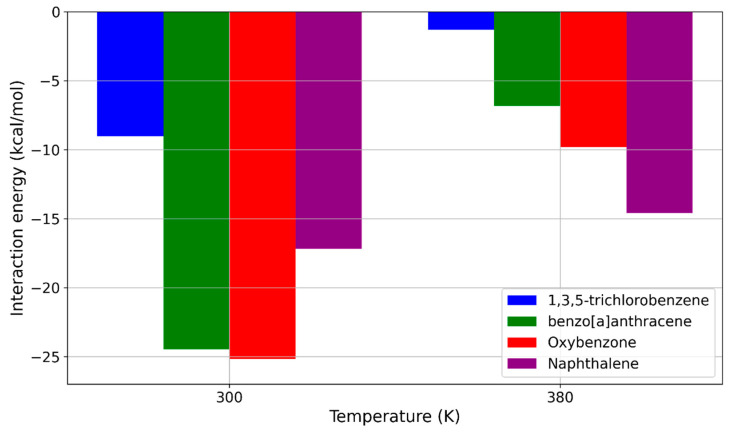
Interaction energies for the remaining sunscreen pollutants at 300 K and 380 K.

The increase in temperature from 300 K to 380 K also led to a significant reduction in interaction energies for the remaining sunscreen pollutants when interacting with PDMS. These results are presented in Figure 9.

**Figure 10:**
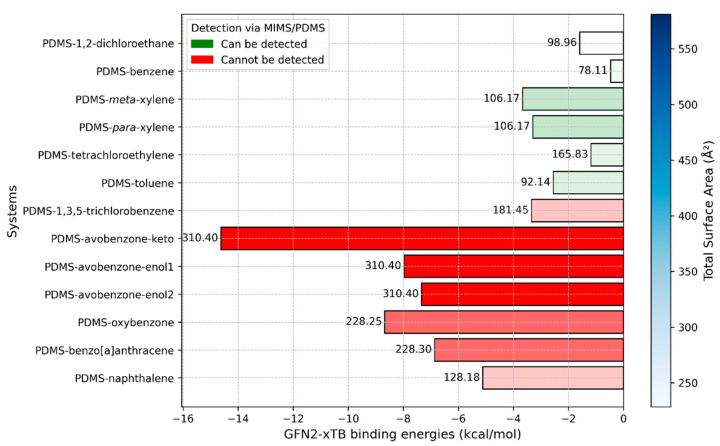
Binding energies between the PDMS chain and selected molecules obtained with the GFN2-xTB method. The numbers on top of the bins represent molecular weight. The intensity of the blue color in the legend indicates the value of the total surface area and is directly correlated to the color intensities of bins. Red bins are related to compounds that current MIMS devices cannot detect, while green bins are related to compounds that can be detected.

The results presented in Figure 9 suggest that the temperature increase from 300 K to 380 K could be a key factor for the application of pure PDMS membranes in MIMS devices for filtering sunscreen molecules. According to our MD simulations, this temperature rise effectively reduced the interaction energy for nearly all the studied sunscreen molecules below the potentially critical threshold of around −10 kcal/mol, as established in Section 2.2. Among the molecules shown in Figure 10, the most significant decrease in interaction energy was observed for benzo[a]anthracene, where the interaction energy dropped from −24.474 kcal/mol to −6.818 kcal/mol. A similar reduction occurred for oxybenzone, where the interaction energy decreased from −25.169 kcal/mol to −9.796 kcal/mol. The already weak interaction energy of 1,3,5-trichlorobenzene further decreased from −9.029 kcal/mol to −1.322 kcal/mol. At 380 K, the interaction energy for naphthalene remained relatively close to its value at 300 K, decreasing only slightly from −17.193 kcal/mol to −14.578 kcal/mol.

All of these results regarding the influence of temperature suggest that this parameter could be critical for the practical application of PDMS-based membranes in MIMS devices. In almost all cases, increasing the temperature to 380 K reduced the interaction energy below the critical threshold of −10 kcal/mol, as determined from the values at 300 K. The only exception was the naphthalene molecule, where the interaction energy remained slightly above −10 kcal/mol. However, given the very small size of naphthalene, it is likely that this reduction is sufficient, as the molecule’s small size and flexibility may allow it to move more easily within the PDMS polymer medium.

### 2.4. GFN2-xTB Ground State Geometries and Binding Energies

In this study, the binding interactions between PDMS and the selected molecules were also investigated using the modern semi-empirical method, GFN2-xTB. Given the size of the studied systems, which consist of approximately 200 atoms each, and the highly flexible nature of polymer chains, we opted for this fast quantum mechanical approach. GFN2-xTB offers a reasonable balance between computational speed and accuracy, making it well-suited for systems of this scale. The binding energies between the PDMS chain and the molecules are presented in Figure 10.

The results presented in Figure 10 are consistent with experimental observations. Specifically, all molecules that are currently undetectable by MIMS exhibit significantly stronger binding energies with PDMS. As expected, the avobenzone molecule—particularly in its keto form—displays the strongest binding with the PDMS chain. However, it is noteworthy that the enol forms of avobenzone have much weaker binding energies compared to the keto form. Similar to the MD simulation results, the two weakest interacting molecules that remain undetectable by MIMS are 1,3,5-trichlorobenzene and naphthalene.

The computationally efficient GFN2-xTB calculations not only allowed us to align our findings with experimental data but also enabled us to obtain optimized structures and further investigate noncovalent interactions using higher-level theoretical methods. In this study, we focused on the interaction between the different forms of avobenzone and PDMS to explain why the enol form exhibits weaker interactions with PDMS compared to the keto form. To achieve this, we conducted an analysis of electron density following the approach described in references [37,38,39], revealing key information about the noncovalent interactions between PDMS and the avobenzone forms, which we discuss in detail in the next section.

Before discussing the strengths of the noncovalent interactions, there is a key geometric aspect that explains why the enol forms do not interact as strongly with PDMS. As shown in Figure 4d, the keto form of avobenzone exhibits a noticeable twist in the middle of the molecule. In contrast, the enol forms are dominated by a strong intramolecular hydrogen bond in the middle, which prevents the molecule from twisting (Figure 4e,f). This rigidity in the enol forms restricts their flexibility when interacting with PDMS. Additionally, the oxygen atom in the central part of the avobenzone molecule is engaged in hydrogen bonding with the OH group, limiting its ability to interact with PDMS. Therefore, the rigidity of the enol forms reduces their ability to adjust their geometry to optimize interactions with the flexible PDMS chains, resulting in weaker overall noncovalent interactions.

This allows us to explain the influence of temperature. Namely, as the temperature increases, the thermal motion of both the PDMS chains and the avobenzone molecule intensifies. While the flexible keto form can adjust its conformation to maintain or even enhance its interactions with PDMS at higher temperatures, the more rigid enol form is less adaptable. This results in a greater loss of interaction energy for the enol form, as the flexible PDMS chains may no longer align as effectively with the relatively fixed geometry of the enol form, leading to a more pronounced reduction in interaction strength.

The twisting of the keto form, combined with the flexibility of the PDMS chains, allows for increased surface contact and thus stronger interactions. In contrast, the rigidity of the enol form and its fixed geometry result in less surface contact with PDMS, especially as thermal motion increases. This lack of adaptability further contributes to the reduction in interaction energy for the enol form, explaining the drop in the interaction energy intensity to 380 K.

### 2.5. Noncovalent Interactions Between PDMS and Avobenzone Forms

**Figure 11:**
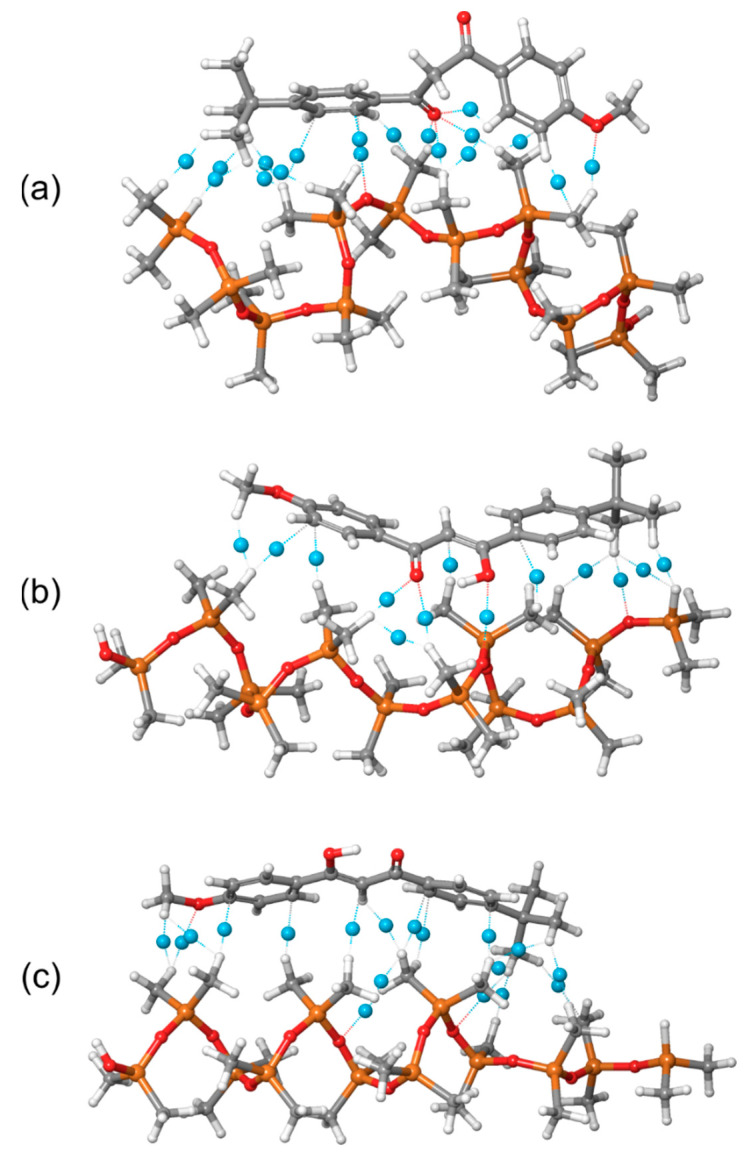
Intermolecular noncovalent interactions between PDMS polymer chain and avobenzone (**a**) keto, (**b**) enol1, and (**c**) enol2 forms (blue dummy atoms are bond critical points).

The significantly greater flexibility of avobenzone’s keto form, compared to the rigidity of the enol form, is also evident in the noncovalent interactions formed with the PDMS polymer chain. The flexibility of the keto form allows for more intensive noncovalent interactions, while the rigid geometry of enol’s form limits its ability to engage in such interactions effectively. These differences in intermolecular noncovalent interactions between PDMS and the various forms of avobenzone are illustrated in Figure 11, highlighting the distinct interaction profiles for each form.

The distribution of noncovalent interactions provides deeper insight into the nature of the interactions between PDMS and avobenzone, helping to explain the calculated MD interaction energies. As previously discussed, both the MD simulations and GFN2-xTB calculations confirmed that the strongest interaction occurs between PDMS and the keto form of avobenzone. This is clearly reflected in Figure 11a, where the keto form exhibits a high number of noncovalent interactions, with the most prominent involving the oxygen atom. Specifically, the oxygen atom in the keto form participates in four significant noncovalent interactions, contributing to its stronger binding energy.

In contrast, the enol1 form shows a significantly lower number of noncovalent interactions, which aligns with its reduced binding energy. The enol2 form, while having a higher number of interactions, lacks any involvement of the crucial oxygen atoms. This absence of oxygen-centered interactions in the enol2 form results in the weakest overall binding energy, underscoring the importance of oxygen atoms in forming strong noncovalent interactions with the PDMS polymer.

## 3. Computational Details

The individual structures of the considered PAHs and the PDMS polymer chain, consisting of 10 monomer units, were first optimized using the semi-empirical GFN2-xTB method, developed by Prof. Grimme and coworkers [40,41,42,43,44]. The optimized structures were then used to generate intermolecular systems for studying the binding energies between PDMS and the considered PAHs. These systems consisted of one PDMS chain and one PAH placed above various locations along the PDMS chain. For each of the intermolecular systems, binding energy was calculated according to the following equation:

(1) $$E_{b}=E_{tot}\left(PDMS+PAH\right)-E\left(PDMS\right)-E\left(PAH\right)\;$$

where $E_{tot}\left(PDMS+PAH\right)$ denotes the total energy of the optimized intermolecular system consisting of a PDMS polymer chain and PAH molecule, E(PDMS) denotes the total energy of the optimized PDMS polymer chain, while E(PAH) denotes the total energy of a PAH molecule.

GFN2-optimized structures of PAH were re-optimized at DFT level, with the B3LYP [45,46,47,48] functional and 6-31G(d,p) basis set, followed by single point energy calculations with the M06-2X [49,50,51,52] density functional, but with 6-311++G(d,p) basis set, in order to obtain information about fundamental quantum molecular descriptors and study noncovalent interactions.

GFN2-xTB calculations were performed as implemented in the xtb 6.7.1. program. GFN2-xTB calculations were run within the atomistica.online molecular modeling platform [34,35], freely available at https://atomistica.online (accessed on 29 August 2024). DFT calculations were performed with the Jaguar program [53,54,55,56], as implemented in the Schrödinger Materials Science Suite 2022-2.

## 4. Conclusions

This study demonstrates the critical role of molecular interactions between PDMS and sunscreen molecules (oxybenzone, naphthalene, benzo[a]anthracene, avobenzone, 1,3,5-trichlorobenzene), providing a foundation for improving PDMS-based membranes in MIMS devices. We first used MD simulations to identify the critical interaction energy needed for efficient filtration, confirming that interaction energies near −10 kcal/mol (at the used level of theory) might be essential for the practical application of PDMS membranes.

Using a combination of molecular dynamics simulations and GFN2-xTB calculations, we established that the interaction energies of sunscreen molecules, particularly avobenzone, are strongly influenced by both molecular geometry and temperature. The keto form of avobenzone exhibited the strongest interactions with PDMS, driven by its flexibility and the involvement of oxygen atoms in noncovalent interactions. In contrast, the enol forms, with their rigid geometries, displayed significantly weaker interactions.

Importantly, our results show that increasing the temperature to 380 K drastically reduces the interaction energies of most sunscreen molecules, bringing them below the critical threshold of −10 kcal/mol. This suggests that temperature modulation could be a viable strategy for enabling the filtration of sunscreen pollutants through PDMS membranes. The findings of this study offer valuable insights into the design of new sorbent materials, highlighting the potential of PDMS-based membranes for the effective separation and detection of sunscreen compounds in MIMS systems.

## Figures and Tables

**Table 1:** Detectable and undetectable sunscreen molecules.

Detectable by Current MIMS	Undetectable by Current MIMS
1,2-dichloroethane	1,3,5-trichlorobenzene
Benzene	Avobenzone and his keto and enol forms
Xylene (*meta* and *para*)	Benzo[a]anthracene
Toluene	Naphthalene
Tetrachloroethylene	Oxybenzone

## Data Availability

The original contributions presented in the study are included in the article/Supplementary Material. Further inquiries can be directed to the corresponding authors.

## Supplementary Materials

The following supporting information can be downloaded at: https://www.mdpi.com/article/10.3390/molecules29204908/s1, Figure S1. Optimized structures of considered pollutants (a) 1,3,5-trichlorobenzene, (b) naphthalene, (c) benzo[a]anthracene, (d) oxybenzone and (e) avobenzone.
