# Supplementary material for: Computational Multiscale Study of the Interaction Between the PDMS Polymer and Sunscreen-Related Pollutant Molecules
Source: Molecules. 2024 Oct 17;29(20):4908. doi: 10.3390/molecules29204908 (PMC11510613; doi:10.3390/molecules29204908)
Supplement: Supplementary file 1 [file molecules-29-04908-s001.zip › molecules-3235112-supplementary.pdf]

Computational Multiscale Study of the Interaction Between the PDMS Polymer and Sunscreen-Related Pollutant Molecules

Stevan Armaković [1,*], Đorđe Vujić [2] and Boris Brkić [2,*]

[1] University of Novi Sad, Faculty of Sciences, Department of Physics, Trg D. Obradovića 4, 21000 Novi Sad, Serbia
[2] BioSense Institute, University of Novi Sad, Dr Zorana Djindjića 1, 21000 Novi Sad, Serbia; d.vujic@biosense.rs
* Correspondence: stevan.armakovic@df.uns.ac.rs (S.A.); boris.brkic@biosense.rs (B.B.)

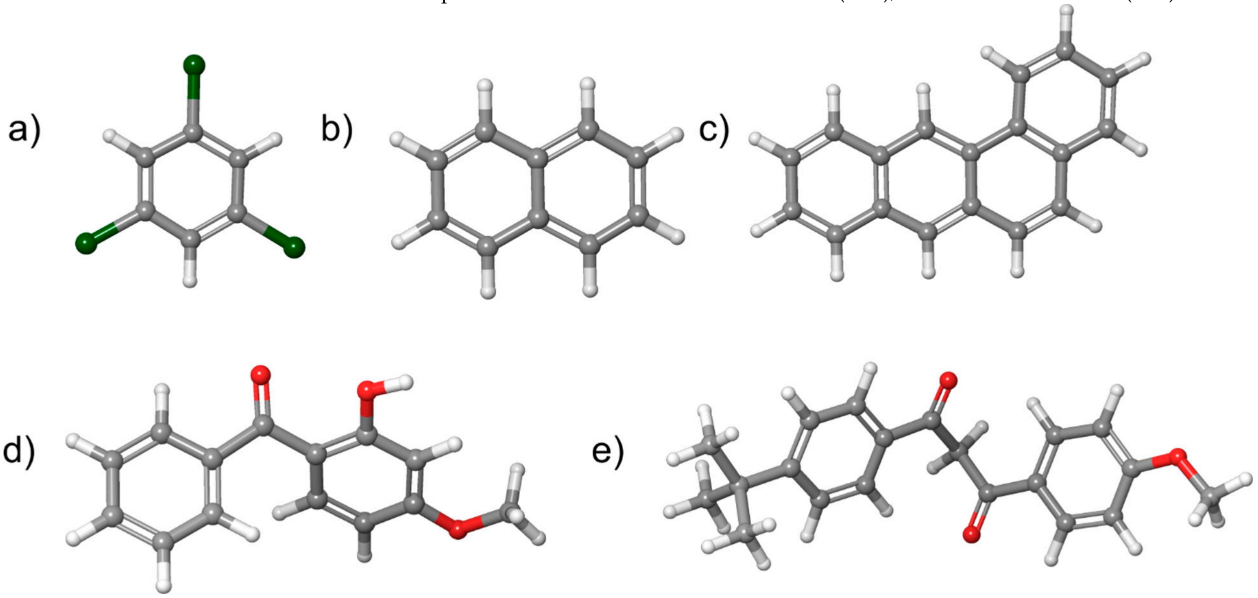

Figure S1. Optimized structures of considered pollutants a) 1,3,5-trichlorobenzene, b) naphthalene, c) benzo[a]anthracene, d) oxybenzone and e) avobenzone
